# Magnetic Field Gradient-Based EKF for Velocity Estimation in Indoor Navigation

**DOI:** 10.3390/s20205726

**Published:** 2020-10-09

**Authors:** Makia Zmitri, Hassen Fourati, Christophe Prieur

**Affiliations:** Département Automatique, Université Grenoble Alpes, CNRS, Grenoble INP, GIPSA-lab, F-38000 Grenoble, France; makia.zmitri@grenoble-inp.fr (M.Z.); christophe.prieur@gipsa-lab.fr (C.P.)

**Keywords:** indoor navigation, magnetic field gradient, spatial derivatives, inertial velocity estimation, Extended Kalman Filter

## Abstract

This paper proposes an advanced solution to improve the inertial velocity estimation of a rigid body, for indoor navigation, through implementing a magnetic field gradient-based Extended Kalman Filter (EKF). The proposed estimation scheme considers a set of data from a triad of inertial sensors (accelerometer and gyroscope), as well as a determined arrangement of magnetometers array. The inputs for the estimation scheme are the spatial derivatives of the magnetic field, from the magnetometers array, and the attitude, from the inertial sensors. As shown in the literature, there is a strong relation between the velocity and the measured magnetic field gradient. However, the latter usually suffers from high noises. Then, the novelty of the proposed EKF is to develop a specific equation to describe the dynamics of the magnetic field gradient. This contribution helps to filter, first, the magnetic field and its gradient and second, to better estimate the inertial velocity. Some numerical simulations that are based on an open source database show the targeted improvements. At the end of the paper, this approach is extended to position estimation in the case of a foot-mounted application and the results are very promising.

## 1. Introduction

Nowadays, the interest in indoor positioning has been growing exponentially, as it represents a topic of research for many different applications, such as in health [1,2], sports [3] and military [4]. A wide range of techniques has been investigated to tackle this problem. Some of them require a costly, heavy and pre-installed infrastructure to work (e.g., Wireless Local Area Network (WLAN) [5], Radio Frequency Identification (RFID) [6], etc.). Others rely on more traditional methods, such as computer vision techniques [7], which can be inaccessible in certain situations (smoke in building for instance). The most common solution to the case where the conditions of intervention, and the availability of pre-installed equipment are unknown, is the use of low-cost Inertial Measurement Units (IMUs), composed of inertial and magnetic sensors. It represents a promising key to solve many problems in indoor positioning. Usually, the outputs of IMUs are used to calculate the velocity and position through an integration process, or to determine the orientation (attitude) [8] through a specific fusion. Nevertheless, because of sensors’ biases and noises, these integrations are biased, and then a drift is observed in velocity (integration of linear acceleration) and position (integration of velocity). Numerous techniques have been proposed in the literature to deal with this problem. Some of them depend on a foot-mounted dead reckoning method called Zero-Velocity Update Technique (ZUPT), such as in [9,10], for example. This method allows reducing the integration to small steps between phases where the foot is at rest on the ground (stance phase). The drift in velocity and position is thus reduced, especially for the accelerometer measurements integration, which allows a longer use before the system diverges too far away from the actual position. In that process, the better the velocity, the better its integration to obtain an improved position. Inertial velocity is also important in other applications that are not necessarily related to position estimation. For instance, in [11], drifts in attitude estimation for human and animal motion evaluation are corrected by removing transient accelerations, using a mathematical derivation of velocity measurements coming from a Global Positioning System (GPS) receiver. In the case where GPS measurements are unavailable, or inaccurate, the precision of this proposed approach is degraded. In [12], it is argued that velocity sensors attached to swimming animals are potentially inaccurate. An evaluation is then conducted with an ellipsoidal micro-turbine that is used to measure the through water speed of a dolphin, by being attached to its body using an array of suction cups. The obtained speed measurements suffer from a few drawbacks, such as the inability to track the speed of the disturbed flow when it drops below the turbine stall speed (U≈0.25 m/s). Other works used the velocity, obtained from a GPS receiver only in the case of outdoor navigation, as an important feature for the classification and prediction of transportation modes [13,14].

### 1.1. Context

The main problem under investigation is the velocity estimation in indoor navigation by means of inertial and magnetic sensors. As known, the presence of magnetic perturbations in indoor environments can be very large [15], due to all metals used in buildings (door frames, aluminum windows, etc.) and potentially to the strong electric currents propagating close-by. Now, one might think that these disturbances can only represent a constraint for indoor positioning. This is the case for heading estimation applications, where the magnetic deviation is seen as a perturbation to be rejected. Then, adaptation techniques are developed to compensate its effect [16]. However, these perturbations are not in fact a random noise. On the contrary, they are well structured by physics equations—for instance, Maxwell’s equations [17]. The latter represent the propagation of electromagnetic phenomena. Therefore, it is considered that rich information lies in these disturbances. Nevertheless, most of the magnetic field-based techniques require either a prior mapping [18], or extra equipment, as with magnetic beacons [19]. Besides, the main goal of most of these works is usually trajectory/position reconstruction and not velocity estimation.

An approach that only relies on a 3-axis magnetometer’s array, a 3-axis accelerometer and a 3-axis gyroscope, in the purpose of velocity estimation, was first introduced in [20,21], and further investigated in [22,23,24]. The proposed technique takes advantage of the magnetic field disturbances that are observed indoors, to estimate the inertial velocity. This preserves the main advantages of purely inertial technology: no prior mapping or other information are required. More recently, in [25,26], the authors also use measurements from an array of 3-axis magnetometers to derive a maximum likelihood estimator. In these works, the velocity is not estimated by directly solving (5). Instead, they consider a model parameter estimation problem where the velocity is viewed as a free parameter and is fitted to the observed data.

In the same context, and based on the idea in [20], but with different dynamic models, other authors have shown, in [27,28,29,30], efficient velocity and position estimation results. In fact, they proved that as long as the magnetic field gradient is non-singular, the velocity is observable and there exists a converging non-linear observer that reconstructs it. In these works, the magnetic field gradient is considered as a measured input for the state-space model and the observer. However, this gradient is usually noisy and is subject to singularities. This influences negatively the observability of the proposed models, which leads to estimation errors. Contrarily, in [21], the authors considered that the magnetic field gradient is not available, instead, the gradient is moved to the state vector and is estimated by an observer. Nevertheless, the gradient’s dynamics are modeled by a white noise, which is a questionable choice, to the best of authors knowledge, and can influence the estimation of velocity.

### 1.2. Contribution

This paper presents a solution to improving the inertial velocity estimation. The proposed approach takes advantage of magnetic disturbances, by using a set of spatially distributed magnetometers to monitor the magnetic field and its spatial derivatives (gradient and its first derivative). The considered state-space model in this work also includes a new magnetic field gradient equation, derived to describe its dynamics. An EKF is proposed to better estimate the inertial velocity in a magnetically disturbed environment, from a 3-axis magnetometer’s array, a 3-axis gyroscope and a 3-axis accelerometer. The novelty in the proposed approach is the development of this specific equation to describe the dynamics of magnetic field gradient. This contribution is evaluated by comparing the proposed model to the one in [27], where the magnetic field gradient is not filtered and is rather used as a noisy input. The inputs for the estimation scheme are the spatial derivatives of the magnetic field, from the magnetometer’s array, and a determined attitude via a gradient descent algorithm, from a triad of inertial and magnetic sensors. A notable improvement in the velocity estimation is shown compared to when the noisy magnetic field gradient is measured and used as an input for the EKF, as in [27]. At the end of the paper, we examine the effect of such velocity improvement on the position estimation in the case of a foot-mounted application aided by ZUPT, and the results are very promising.

This paper is organized as follows. In Section 2 some preliminaries and notations are introduced and the principle of the magneto-inertial navigation problem is stated, then the magnetic field gradient dynamic equation is established. An EKF is designed in Section 3, where the gradient equation is added, to tackle measurement noises and to estimate not only the velocity but also the magnetic field and its gradient. The EKF is fed with a determined quaternion, given by an attitude estimation block. In Section 4, the ZUPT-aided position estimation is detailed, based on the previous velocity estimation, in the context of foot-mounted inertial navigation. Section 5 presents a scenario test based on an open source database [31] representing a foot-mounted navigation scheme. The obtained results in this case are displayed. In Section 6, some conclusions and potential future works are stated.

## 2. Problem Formulation

The problem under consideration is how to improve the inertial velocity estimation using only Micro Electro Mechanical Systems (MEMS) inertial sensors, composed of a 3-axis accelerometer and a 3-axis gyroscope, as well as a spatially distributed 3-axis magnetometer’s array. A new state-space model is proposed and its contribution is demonstrated through an EKF-based approach. In the end of the paper, the obtained results of the improved velocity estimation are extended to the position estimation in a foot-mounted framework.

### 2.1. Notation

To address the problem cited above, two frames are used:

- A local inertial frame ${ℜ}_{n}$ fixed to the Earth (Earth rotation is neglected) and its associated orthonormal basis; $B_{n}=\left({\vec{i}}_{n},{\vec{j}}_{n},{\vec{k}}_{n}\right)$;
- A body frame ${ℜ}_{b}$ attached to the moving rigid body and its associated orthonormal basis. $B_{b}=\left({\vec{i}}_{b},{\vec{j}}_{b},{\vec{k}}_{b}\right)$.

Variables expressed in ${ℜ}_{n}$ (resp. ${ℜ}_{b})$ are marked by the subscript *n* (resp. *b*).

Let $R_{b\leftarrow n}\in SO\left(3\right)$ be the rotation matrix between the two frames, from ${ℜ}_{n}$ to ${ℜ}_{b}$. For the sake of simplicity, in the rest of the paper the notation $R_{b\leftarrow n}$ is omitted and is replaced by *R*. This matrix can be expressed in terms of quaternion as follows:

(1) $$R=\left[\begin{array}{ccc} 2(q_{0}^{2}+q_{1}^{2})-1 & 2(q_{1}q_{2}+q_{0}q_{3}) & 2(q_{1}q_{3}-q_{0}q_{2})\\ 2(q_{1}q_{2}-q_{0}q_{3}) & 2(q_{0}^{2}+q_{2}^{2})-1 & 2(q_{0}q_{1}+q_{2}q_{3})\\ 2(q_{0}q_{2}+q_{1}q_{3}) & 2(q_{2}q_{3}-q_{0}q_{1}) & 2(q_{0}^{2}+q_{3}^{2})-1 \end{array}\right]$$

The unit quaternion, denoted by *q*, is a hypercomplex number of rank 4 such that,

(2) $$q={\left[q_{0}\;\;\;q_{vect}^{⊤}\right]}^{⊤}$$

where $q_{0}$ is the scalar part and $q_{vect}={\left[q_{1}\;\;\;q_{2}\;\;q_{3}\right]}^{⊤}$ is the vector part of quaternion. The reader is invited to refer to [32] for more details about quaternion algebra.

The rigid body under consideration can simultaneously translate and rotate in 3D space, and its displacement is represented with the position vector $M_{n}={\left[x_{n}\;\;y_{n}\;\;z_{n}\right]}^{⊤}\in R^{3\times 1}$ in ${ℜ}_{n}$. Then, $v_{n}=\frac{dM_{n}}{dt}={\left[v_{nx}\;\;v_{ny}\;\;v_{nz}\right]}^{⊤}\in R^{3\times 1}$, the inertial velocity vector to be estimated in ${ℜ}_{n}$, and $a_{n}=\frac{dv_{n}}{dt}={\left[a_{nx}\;\;a_{ny}\;\;a_{nz}\right]}^{⊤}\in R^{3\times 1}$ the acceleration vector. Vectors $v_{n}$ and $a_{n}$ can also be expressed in ${ℜ}_{b}$ by simply multiplying them by *R*.

Since inertial and magnetic sensors are used in this framework, then the following variables are considered:

- The angular velocity $\omega^{\frac{b}{n}}={\left[\omega_{x}\;\;\omega_{y}\;\;\omega_{z}\right]}^{⊤}\in R^{3\times 1}$, of ${ℜ}_{b}$ with respect to ${ℜ}_{n}$, measured by a 3-axis gyroscope. The corresponding skew-symmetric matrix is defined such as
  (3) $$\left[\omega^{\frac{b}{n}}\times \right]=\left(\begin{array}{ccc} 0 & -\omega_{z} & \omega_{y}\\ \omega_{z} & 0 & -\omega_{x}\\ -\omega_{y} & \omega_{x} & 0 \end{array}\right)$$
- The acceleration $a_{b}={\left[a_{bx}\;\;a_{by}\;\;a_{bz}\right]}^{⊤}\in R^{3\times 1}$ of ${ℜ}_{b}$, measured by a 3-axis accelerometer;
- The magnetic field $B_{b}={\left[B_{bx}\;\;B_{by}\;\;B_{bz}\right]}^{⊤}\in R^{3\times 1}$, measured in ${ℜ}_{b}$ by a 3-axis magnetometer, which depends on time and space;
- The Jacobian matrix $\nabla B_{b}\in R^{3\times 3}$, which represents the magnetic field gradient, measured on a fixed point $P_{b}={\left[x_{bp}\;\;y_{bp}\;\;z_{bp}\right]}^{⊤}\in R^{3\times 1}$ and defined by

(4) $$\nabla B_{b}\left(P_{b}\left(t\right)\right)=\frac{\partial B_{b}\left(P_{b}\left(t\right)\right)}{\partial P_{b}\left(t\right)}$$

Note here that, using (4) and according to the rule of vector derivative versus vector, the magnetic field gradient $\nabla B_{b}$ should be defined as a 9×1 vector. However, it can also be mapped by a unique bijection to a matrix in $R^{3\times 3}$, which justifies the previous dimension definition.

### 2.2. Magnetic Field and Its Gradient

A rigid body located inside a magnetically disturbed area is considered, which is a situation that is often observed indoors [15]. The disturbances in the magnetic field are useful information in this work. The dynamic of the magnetic field measured in ${ℜ}_{b}$ obeys to the following equation (× is the cross product of two vectors in $R^{3}$):

(5) $$\frac{dB_{b}}{dt}=-\omega^{\frac{b}{n}}\times B_{b}+\nabla B_{b}v_{b}$$

This equation ensures that $v_{b}$ is observable and can be estimated, provided that $\nabla B_{b}$ is non-singular (see [20] for observability proof). Under this assumption, the velocity can be estimated using $\nabla B_{b}$, which enhances the performance of any inertial navigation system, as demonstrated in [21]. However, one of the major remaining difficulties is to reliably measure $\nabla B_{b}$. For this purpose, a spatially distributed magnetometer’s array is considered. This array provides magnetic field measurements that are usually noisy, then, when computing spatial derivatives, this noise can get larger. It follows that $\nabla B_{b}$ is also corrupted by noise. This noise can degrade the velocity estimation, especially when $\nabla B_{b}$ has low values (more precise simulations on the matter are in [33]). A way to tackle this problem is to filter $\nabla B_{b}$. To do so, an equation representing its dynamic should be proposed.

For that, the temporal derivative of $\nabla B_{n}$ in ${ℜ}_{n}$ is introduced, such as

(6) $$\frac{d\nabla B_{n}}{dt}=\frac{d\nabla B_{n}}{dM_{n}}\frac{dM_{n}}{dt}=T_{n}v_{n}$$

where $T_{n}\in R^{3\times 3\times 3}$ is a tensor representing the first spatial derivative of $\nabla B_{n}$ in ${ℜ}_{n}$. To ease the reading, this tensor is alternatively defined as a matrix in $R^{9\times 3}$ through a bijection, and can be represented as follows:

(7) $$T_{n}=\frac{d\nabla B_{n}}{dM_{n}}=\left[\begin{array}{ccc} \nabla \alpha_{11} & \nabla \alpha_{12} & \nabla \alpha_{13}\\ \nabla \alpha_{21} & \nabla \alpha_{22} & \nabla \alpha_{23}\\ \nabla \alpha_{31} & \nabla \alpha_{32} & \nabla \alpha_{33} \end{array}\right]$$

and $\nabla \alpha_{ij}={\left[\frac{\partial \alpha_{ij}}{\partial x_{m}}\;\;\frac{\partial \alpha_{ij}}{\partial y_{m}}\;\;\frac{\partial \alpha_{ij}}{\partial z_{m}}\right]}_{1\leq i,j\leq 3}^{⊤}$, with $\alpha_{ij}$ representing the elements of $\nabla B_{n}$.

The temporal derivative $\frac{d\nabla B_{n}}{dt}$ can also be written, such that

(8) $$\begin{array}{ccc} \frac{d\nabla B_{n}}{dt} & = & \frac{d(R^{⊤}\nabla B_{b}R)}{dt}=\frac{dR^{⊤}}{dt}\nabla B_{b}R+R^{⊤}\frac{d\nabla B_{b}}{dt}R+R^{⊤}\nabla B_{b}\frac{dR}{dt}\\ & = & R^{⊤}\left[\omega^{\frac{b}{n}}\times \right]\nabla B_{b}R+R^{⊤}\frac{d\nabla B_{b}}{dt}R+R^{⊤}\nabla B_{b}\left(-\left[\omega^{\frac{b}{n}}\times \right]R\right) \end{array}$$

From (6) and (8), the following equality is obtained

(9) $$R^{⊤}\frac{d\nabla B_{b}}{dt}R=T_{n}v_{n}+R^{⊤}\nabla B_{b}\left[\omega^{\frac{b}{n}}\times \right]R-R^{⊤}\left[\omega^{\frac{b}{n}}\times \right]\nabla B_{b}R$$

By multiplying both sides of (9) by *R* and $R^{⊤}$, respectively, the following equation is deduced,

(10) $$\frac{d\nabla B_{b}}{dt}=T_{b}v_{b}+\nabla B_{b}\left[\omega^{\frac{b}{n}}\times \right]-\left[\omega^{\frac{b}{n}}\times \right]\nabla B_{b}$$

where $T_{b}$ is the first spatial derivative of $\nabla B_{b}$, with the same form as (7), represented in ${ℜ}_{b}$. The reader can check [34] for more information on how $T_{b}$ is measured.

In Section 3, the dynamic model, specific to the studied problem, is expanded by including (10), and an EKF is designed to filter $\nabla B_{b}$ to improve the velocity estimation.

## 3. Inertial Velocity, Magnetic Field and Magnetic Field Gradient Estimation

**Figure 1:**
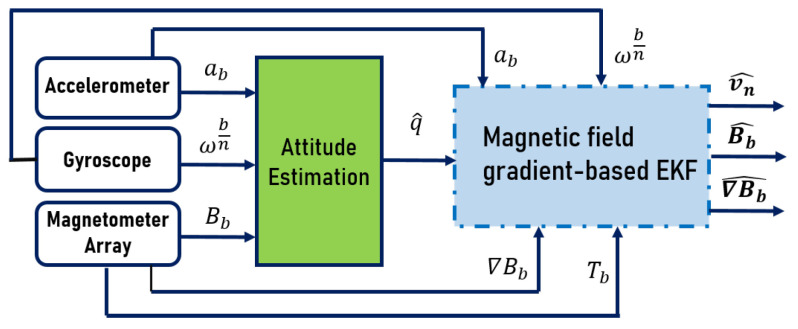
Overall diagram for estimation.

This section is focused mainly on the inertial velocity estimation, by using an IMU and an array of spatially distributed magnetometers. A block diagram of the proposed approach is shown in Figure 1. The main novelty resides on the blue block, which represents a magnetic field gradient-based EKF for estimating not only the inertial velocity $\hat{v_{n}}$, but also the magnetic field $\hat{B_{b}}$, and its gradient $\hat{\nabla B_{b}}$. This EKF is fed with an estimated quaternion $\hat{q}$, given by the green block, that depicts a gradient descent attitude estimation algorithm [35]. The two blocks are explained in detail in the following sub-sections.

### 3.1. Magnetic Field Gradient-Based Ekf

A magnetic field gradient-based EKF is proposed based on a 3-axis magnetometer’s array, a 3-axis gyroscope and a 3-axis accelerometer. The continuous-time dynamic model used to establish the EKF can be written, such that

(11) $$\left\{\begin{array}{c} \;\frac{dv_{n}}{dt}=R{\left(\hat{q}\right)}^{⊤}a_{b}-g\\ \;\frac{dB_{b}}{dt}=-\omega^{\frac{b}{n}}\times B_{b}+\nabla B_{b}R\left(\hat{q}\right)v_{n}\\ \frac{d\nabla B_{b}}{dt}=T_{b}R\left(\hat{q}\right)v_{n}+\nabla B_{b}\left[\omega^{\frac{b}{n}}\times \right]-\left[\omega^{\frac{b}{n}}\times \right]\nabla B_{b} \end{array}\right.$$

The state vector for this dynamic state-space model is $x={\left[v_{n}\;\;B_{b}\;\;\nabla B_{b}\right]}^{⊤}\in R^{11\times 1}$, the input vector is $u={\left[\hat{q}\;\;\omega^{\frac{b}{n}}\;\;a_{b}\;\;T_{b}\right]}^{⊤}\in R^{17\times 1}$, and the output (measurement) vector is $y={\left[B_{b}\;\;\nabla B_{b}\right]}^{⊤}\in R^{8\times 1}$. Recall that 7 elements of $T_{b}$ are sufficient to calculate all the tensor’s components [34]. The matrix $R(\hat{q})$ is defined in (1), where $\hat{q}$ is the estimated quaternion. Note that the term $v_{b}$ in (5) and (10) is replaced by $R\left(\hat{q}\right)v_{n}$ since the inertial velocity needs to be estimated in ${ℜ}_{n}$ rather than in ${ℜ}_{b}$.

**Figure 2:**
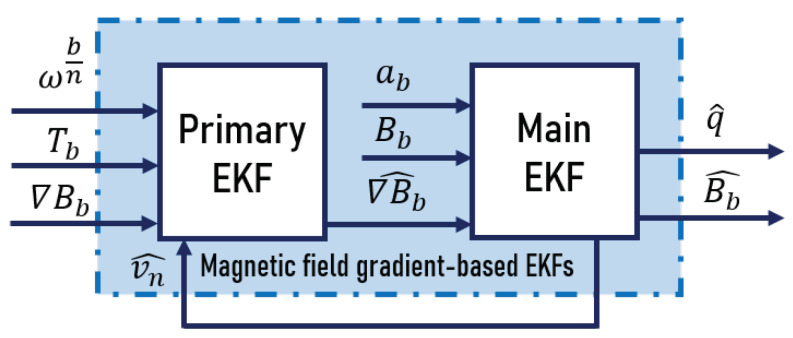
Magnetic field gradient-based EKFs [34].

The magnetic field measurements are usually noisy; then, when extracting higher order derivatives (in this case $\nabla B_{b}$), this noise becomes more important, due to the different approximations taken into account in some numerical computations. It follows that $\nabla B_{b}$ is also affected by noise. This can cause unbounded velocity estimation errors, especially when $\nabla B_{b}$ has low values. For this reason, filtering $\nabla B_{b}$ instead of using it directly as an input, corrupted with noise in the EKF, improves the velocity estimation. As $T_{b}$, defined in (10), is measurable, it is possible to add $\nabla B_{b}$ to *x*. A first schema of the magnetic field gradient-based EKF was presented in [34]. The estimation approach was based on two EKFs, in cascade, as displayed in Figure 2. The primary EKF used the third equation in (11) as a dynamic model, while the main EKF used the first and second equations in (11). In addition to these two equations, the main EKF also estimated quaternion by including (13) in its model.

**Figure 3:**
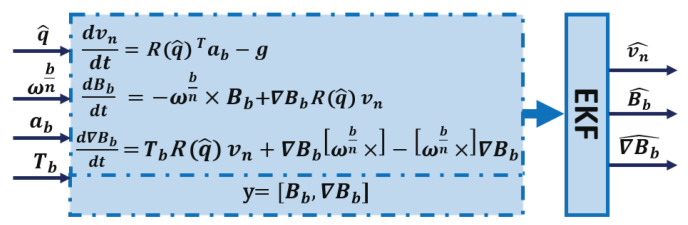
Magnetic field gradient-based EKF.

To go further in this paper, we propose to simplify the estimation architecture in Figure 2, by using the compact dynamic model (11). The general schema of estimation is presented in Figure 3, where a single EKF is rather used.

It is important to indicate that, unlike [34], in the new proposed model (11), Equation (13) representing the quaternion dynamics is discarded. Instead, attitude estimation is conducted using the gradient descent algorithm represented with the green block in Figure 1. The reason for this choice is to avoid complexity. By representing the proposed approach through different blocks (EKF and attitude gradient algorithms), it is possible to further investigate the velocity estimation problem, independently from the attitude one, and vice versa. This allows the user to efficiently test and compare different velocity estimation models. In fact, the effect of attitude estimation can vary from one model to another (if it is included in a single EKF), which makes it impossible to conclude on the advantage of the proposed velocity estimation approach, as the results are also influenced by the varying errors on the attitude estimate (from one model to another and also between static and dynamic cases in motion), and the correlation in the covariance matrix. Furthermore, by adding the magnetic field gradient equation to the main EKF of [34], and eliminating the primary EKF, the process covariance matrix dimension increases from $Q\in R^{10\times 10}$ to $Q\in R^{15\times 15}$. This makes it harder to properly tune *Q*, especially when taking into account the correlation between all the variables (it is brought to the attention of the reader that the magnetic field gradient equation is dependent on all the other variables in the model, and mainly *q*). This means that the off-diagonal elements of *Q* should not be set to zero (which is generally the case by assuming there are no correlations between variables’ noises). Then, all elements of *Q* should be properly chosen, which makes the task very complicated. In this work, states’ noise dependencies are neglected and the off-diagonal elements of *Q* are set to zero. The remaining values are then concluded from a trial and error evaluation, as follows: $Q_{i,j}={\left\{0.01^{2}\right\}}_{1\leq i=j\leq 3}$ and $Q_{i,j}={\left\{0.001^{2}\right\}}_{4\leq i=j\leq 11}$. In the purpose of evaluating and highlighting the contribution of adding the magnetic field gradient equation to the main model of [34] (by eliminating the primary EKF of [34]), without being biased by other variables effect (the estimation of *q*), both these processes are separated.

The two models for process and measurements in Figure 3 can be represented by the following general nonlinear form:

(12) $$\begin{array}{c} \begin{array}{cc} x[k]= & f(x[k-1],u[k],\nu [k])\\ y[k]= & h(x[k],u[k],\eta [k]) \end{array} \end{array}$$

where x[k] is the state vector at time step *k*, y[k] is the measurement vector, u[k] is the input, f(.) is a nonlinear function that represents the state-space model, h(.) is a nonlinear function that represents the measurement model, and ν[k] and η[k] are the process and measurement noises, respectively, and are assumed to be zero-mean, white, Gaussian and uncorrelated. Note that, in order to determine f(.) and h(.), a discretization procedure that transforms the continuous-time equations in Figure 3 into a discrete-time model must be undertaken. The Runge-Kutta fourth order method [36] is used for the discretization.

### 3.2. Quaternion Estimation

The kinematic equation describing the variation in rigid body’s attitude, in terms of quaternion, can be defined from angular velocity measurements given by a 3-axis gyroscope, such as,

(13) $$\frac{dq}{dt}=\frac{1}{2}\left[\omega_{q}\times \right]q=\frac{1}{2}\left(\begin{array}{cccc} 0 & -\omega_{x} & -\omega_{y} & -\omega_{z}\\ \omega_{x} & 0 & \omega_{z} & -\omega_{y}\\ \omega_{y} & -\omega_{z} & 0 & \omega_{x}\\ \omega_{z} & \omega_{y} & -\omega_{x} & 0 \end{array}\right)q$$

where $\omega_{q}={\left[0\;\;\omega^{\frac{b}{n}⊤}\right]}^{⊤}\in R^{4\times 1}$, the quaternion form of angular velocity, and $\left[\omega_{q}\times \right]$ is its skew-symmetric matrix. However, the gyroscope has a long-term drift which is due to noise and bias. So, by simply integrating (13), a drift can be observed on quaternion. The most common solution for such a problem is to use a data fusion approach that merges measurements coming from gyroscopes, accelerometers, and magnetometers. The main methods are based on Kalman filters (KFs) [37], Extended Kalman filters (EKFs) [38], complementary filters [35,39,40], or observers [41]. Nevertheless, one should keep in mind the problem of magnetic disturbances in indoor navigation. These perturbations are known to affect the precision of most attitude determination techniques, which calls for approaches that investigate this case, such as in [35,38,41].

In [35], the authors proposed a new algorithm that uses inertial and magnetic measurements to provide a precise attitude estimation through incorporating magnetic distortion and gyroscope drift compensations. The main idea is to use 3-axis accelerometer and 3-axis magnetometer measurements in an analytically derived and optimized gradient descent algorithm, in order to compute the direction of gyroscope measurement error as a quaternion derivative. This algorithm is computationally inexpensive, as it requires 277 scalar arithmetic operations each update step, it is efficient at low sampling rates and it has only two adjustable parameters defined by observable system characteristics. Moreover, it eliminates the need for the reference direction of Earth’s magnetic field to be predefined. Then, in what follows, this algorithm is implemented (green block in Figure 1) to determine $\hat{q}$.

## 4. Position Estimation in the Context of Foot-Mounted Inertial Navigation

**Figure 4:**
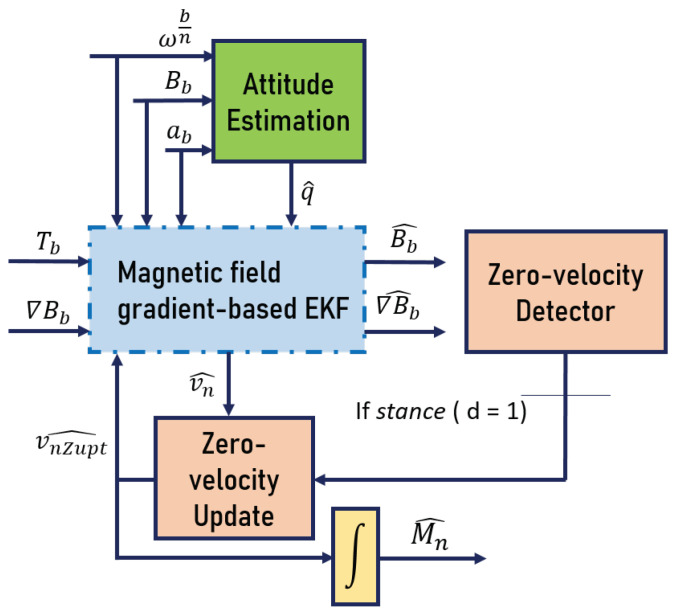
ZUPT-aided position estimation.

In this section, we examine the effect of such velocity estimation improvements on the position one, with a focus on a foot-mounted navigation framework. The proposed algorithm is a combination between the magnetic field gradient-based EKF and ZUPT. The general schema of estimation is presented in Figure 4. The right red block represents the zero-velocity detector, denoted by *d*. In the case where d=1, a zero-velocity update (left red block) is applied on the estimated inertial velocity $\hat{v_{n}}$ resulting from the blue block. The updated velocity $\hat{v_{nZupt}}$ is fed to the yellow block for integration, in order to obtain the position $\hat{M_{n}}$. The red blocks are described in the following subsections.

### 4.1. Zero-Velocity Detector

The objective of a zero-velocity detector is to decide whether, during a time epoch that consists of W∈N observations (i.e., window size) between the time instants *l* and l+W−1, the IMU is moving or stationary, given the measurements $a_{b}$ and $\omega^{\frac{b}{n}}$. At each sample, this detector, denoted *d*, can have one of two values: d=1, which corresponds to the stance phase (the entire period during which the foot is on the ground) or d=0, which represents the swing phase (the entire period during which the foot is in the air for limb advancement). Mathematically, this detection process can be seen as a binary hypothesis testing problem, where the detector indicates that the IMU is stationary (i.e., d=1) if,

(14) $$T_{s}\left(a_{b},\omega^{\frac{b}{n}}\right)\leq \gamma$$

with $T_{s}\left(a_{b},\omega^{\frac{b}{n}}\right)$, the test statistics of the detector and γ, the detection threshold.

The test statistics can have multiple forms, depending on the chosen detector. In related works, different detectors have been evaluated [10] from those depending only on accelerometer data (such as Acceleration Moving Variance Detector and Acceleration Magnitude Detector), to those that are angular rate-based (Angular Rate Energy Detector), or even pressure measurements [42]. In this paper, the Stance Hypothesis Optimal Detector (SHOE) [43] is chosen, as it represents a combination between acceleration and angular rate-based detectors, and has been proven to outperform other detectors in the literature for its robustness to changes in gait speed as well as its high positional accuracy. Concretely, SHOE computes $T_{s}\left(a_{b},\omega^{\frac{b}{n}}\right)$ in the following way:

(15) $$T_{s}\left(a_{b},\omega^{\frac{b}{n}}\right)=\frac{1}{W}\sum_{k=l}^{l+W-1}(\frac{1}{\sigma_{a}^{2}}\left|\right|a_{b,k}-g\frac{{\bar{a}}_{b,l}}{\left|\right|{\bar{a}}_{b,l}\left|\right|}{\left|\right|}^{2}+\frac{1}{\sigma_{\omega }^{2}}\left|\right|{\omega_{k}}^{\frac{b}{n}}{\left|\right|}^{2})$$

where *W* is the window size (the number of sensor readings), $\sigma_{a}^{2}$, $\sigma_{\omega }^{2}$ are the variances of the acceleration and angular rate measurements, ${\bar{a}}_{b,l}$ denotes the mean over *W* samples, and *g* is the gravity.

### 4.2. Zupt

If the detector *d* has declared the stationary case (i.e., d=1), $\hat{v_{n}}$ should give a zero-velocity estimate. However, due to diverse errors, it most likely will not. This motivates the use of ZUPT, as it corrects these drifts, which greatly improves the velocity estimation, as shown in the literature. If d=1 at time *k*, the actual value of inertial velocity is assumed to be known, and then its estimate $\hat{v_{n}}$ is reset to zero. This is actually done inside the EKF, in a way where the velocity estimate $\hat{v_{n}}$ is constantly corrected. In Figure 4, the ZUPT-based velocity estimate is represented with $\hat{v_{nZupt}}$. Consequently, by updating the velocity estimate, a better position estimation should be obtained after integrating $\hat{v_{nZupt}}$ (yellow box in Figure 4).

## 5. Simulations and Results

In this section, the performance of the proposed magnetic field gradient-based EKF is displayed. The improvements in inertial velocity estimation are highlighted when $\nabla B_{b}$ is filtered. In the end, we examine the effect of such an improvement on the position in a foot-mounted navigation framework aided by ZUPT.

### 5.1. Groundwork for Simulations

One of the most common problems in pedestrian navigation is the knowledge of ground truth, as it enables us to compare proposed algorithms and contributions with references. In [31], authors simulate a trajectory (position and attitude), that is based on a real human walk pattern. Synthetic noiseless IMU data are provided, sampled at a frequency of 100 Hz. A set of signals from a spatially distributed magnetometers array is also considered (one signal is given by [31] and the others are simulated accordingly). The reader can refer to this website—https://lopsi.weebly.com/downloads.html—to download one of the proposed data sets corresponding to ground truth trajectories, and to have more details about the different chosen parameters. In this simulation, a closed 3-loop trajectory in rectangular path of 12 m×7 m is used to represent the ground truth. Then, an additive random zero-mean white Gaussian noise is added, as detailed in Table 1.

### 5.2. Main Results

#### 5.2.1. Attitude Estimation Results

**Figure 5:**
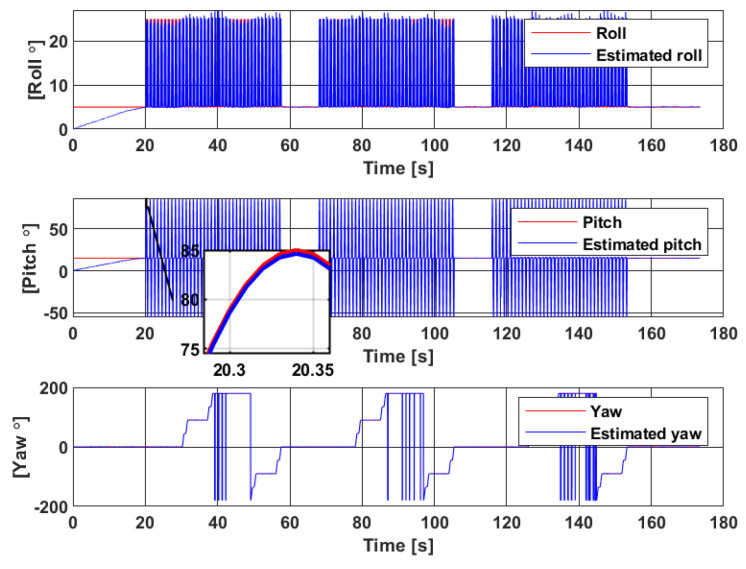
Euler angle estimation through Madgwick filter [35].

To determine the body attitude in quaternion, Madgwick’s gradient descent algorithm [35] is used, as it has been proven robust to magnetic disturbances. The constant β=0.008 (divergence rate) is fixed through a trial and error scheme and by taking into account gyroscope measurement errors. The estimated quaternion $\hat{q}$ is used to calculate the rotation matrix $R(\hat{q})$ through (1). This matrix is important in velocity estimation as it is used in the model in Figure 3. The estimated quaternion is converted into Euler angles, as shown in Figure 5.

The estimated Euler angles converge in less than 20s despite initializing the EKF with values that are different from the true ones. Moreover, the filter is robust against the high standard deviation noise added to magnetic measurements. However, some jumps are seen on the *yaw* estimation when the angle reaches 180°. This is a common problem of discontinuity in Euler angle’s representation. Additionally, it is assumed that the direction of gravity defines the vertical *z*-axis of the IMU. The *yaw* angle represents the rotation around this axis. This means that any error in *yaw* estimation will then generate errors in position reconstruction along the *z*-axis.

#### 5.2.2. Magnetic Field Gradient-Based EKF Results

**Figure 6:**
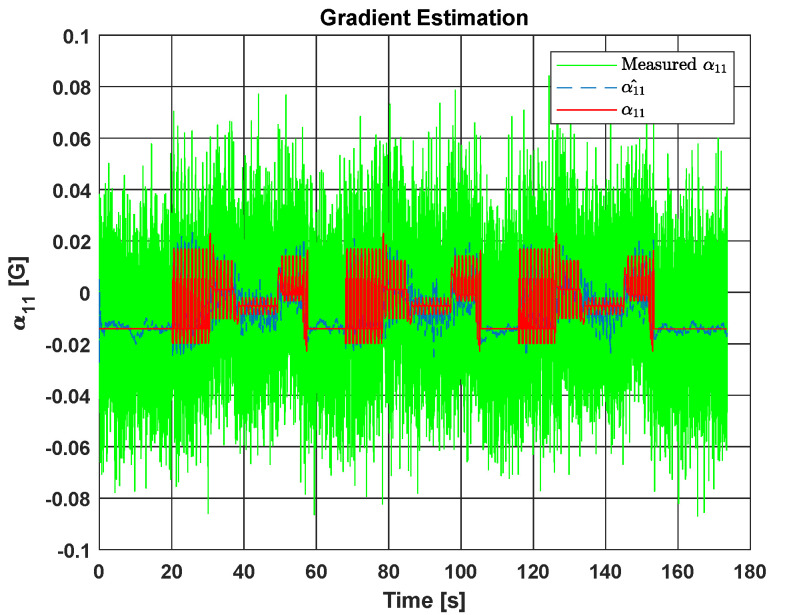
Estimation of the first element $\alpha_{11}$ of $\nabla B_{b}$.

As proposed earlier in this paper, $\nabla B_{b}$ should be filtered from noise, for the purpose of better estimating the inertial velocity. Figure 6 displays the estimation results for the first element $\alpha_{11}$ of $\nabla B_{b}$. The estimated gradient (in blue dashed line) is close to the theoretical one (in red solid line) even though the initialization values are different from the ground-truth ones.

**Figure 7:**
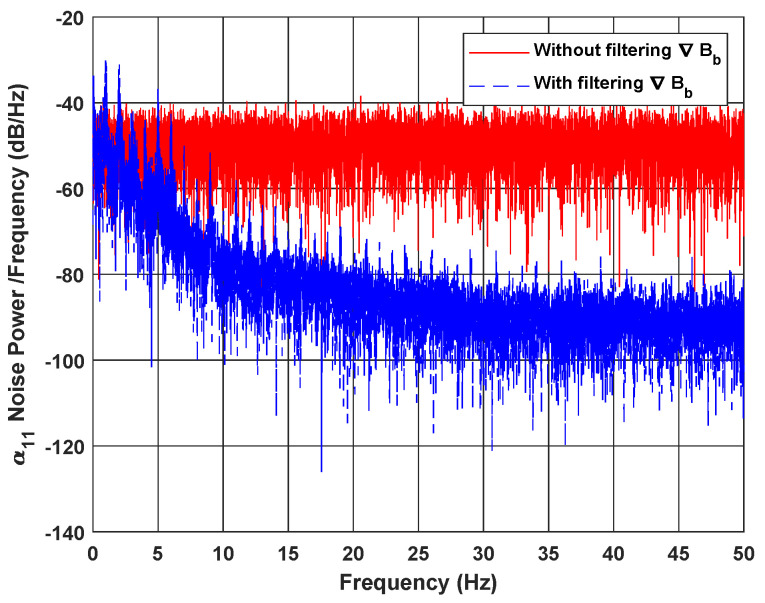
$\alpha_{11}$ noise PSD with and without filtering $\nabla B_{b}$.

Let $\eta_{\alpha_{11}}$ represent the noise of the first element $\alpha_{11}$ of $\nabla B_{b}$. In Figure 7, the Power Spectral Density (PSD) [45] of this noise is presented, before and after filtering $\nabla B_{b}$ with the proposed EKF. This metric represents the square of Fourier transformation module, divided by the spectral bandwidth. It basically describes how the power of a signal is distributed over frequency, which is an interesting criterion to evaluate the noise compensation.

Figure 7 shows that, in the case of filtering $\nabla B_{b}$ (by adding (10) to (11)), the noise power of its elements (e.g., $\alpha_{11}$ in this case), represented in blue dashed line, is lower than that obtained without the filtering process (when $\nabla B_{b}$ is not in the state vector), represented in red solid line, and it decreases continuously along the frequency range. The mean of noise PSD error between both cases is around ≈−29.77dB, which justifies the effectiveness of the proposed approach. Another way that is used to quantify noise in a signal is by computing the Signal to Noise Ratio (SNR) [45], which is the ratio of the power of true signal $\alpha_{11}$ to the power of its noise $\eta_{\alpha_{11}}$. The SNR of $\alpha_{11}$ increases from $SNR_{without}=-9.46\;\mathrm{dB}$ when $\nabla B_{b}$ is not filtered, to $SNR_{with}=-0.42\;\mathrm{dB}$, when it is done. This proves, again, that $\nabla B_{b}$ noise is greatly reduced with the proposed model and filter.

**Figure 8:**
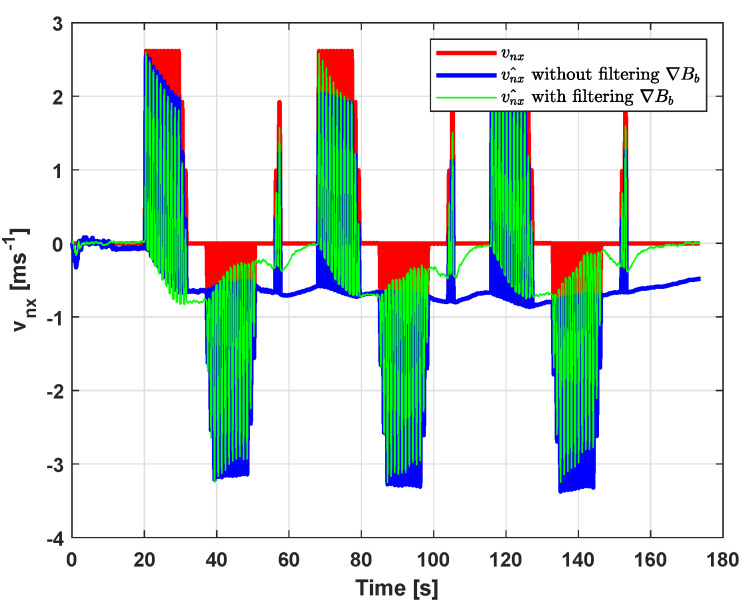
Estimation of $v_{nx}$ with and without filtering $\nabla B_{b}$.

The advantage of this filtering process is also observed during the velocity estimation, as shown in Figure 8, where the *x* axis component of the inertial velocity is plotted. The velocity estimate $\hat{v_{nx}}$ (green solid line) given by the proposed approach is closer to the ground truth velocity (red solid line) than when $\nabla B_{b}$ is used as a noisy input (blue solid line).

As indicated in Table 2, the RMSE between the estimated velocity $\hat{v_{n}}$ and the true one $v_{n}$ is $0.37\;{\mathrm{ms}}^{-1}$ for the case where the proposed approach is not applied, versus $0.27\;{\mathrm{ms}}^{-1}$ when it is done. This improvement is beneficial in some applications that require measuring the velocity with a certain precision. The performance of the magnetic field gradient-based EKF is also compared to the first work [34] and better results are shown in terms of velocity RMSE (and eventually position; see Table 3). This presents one of the contributions of the paper and it demonstrates the innovation compared to [34]. It is underlined here that this improvement is obtained after careful tuning of the state and measurement noise covariance matrices of the proposed EKF. From Table 2, it can be seen that the main contribution of this work resides on the yellow colored line, where the smallest value of RMSE compared to the other approaches is observed.

#### 5.2.3. Application: Extending to Position Estimation

**Figure 9:**
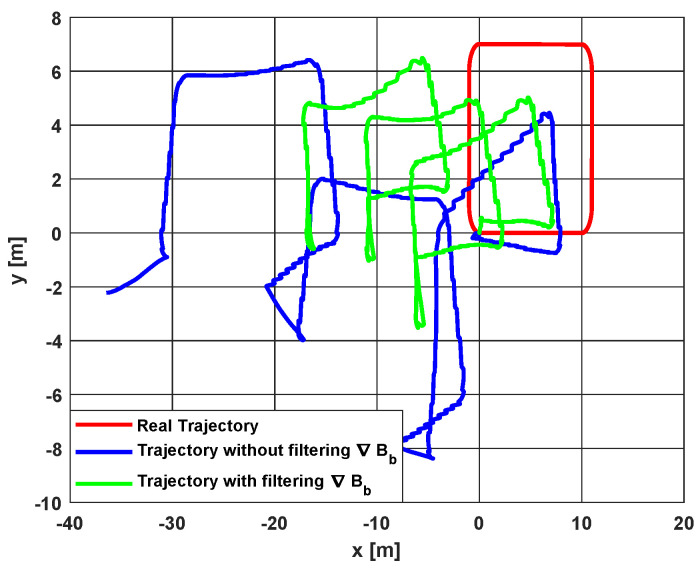
2D trajectory reconstruction with and without filtering $\nabla B_{b}$.

One possible application, that highlights the importance of the decrease in the velocity estimation error, is position reconstruction through an integration of $\hat{v_{n}}$ (without ZUPT). Note here that the main objective of this work is improving velocity estimation, which is demonstrated in Table 2. Nevertheless, to validate the proportionality between velocity determination and position reconstruction, the following example is considered. The impact can be seen by plotting the 2D representation of the estimated trajectory in Figure 9. A noticeable drift compensation is observed when $\nabla B_{b}$ is filtered. Indeed, the slightest improvement in velocity estimation can largely affect the reconstruction of trajectory, as less errors are generated, and thus there is less accumulation during the integration process. It is observed that there is a jump after each loop for both the green and the blue plots in Figure 9. The jumps in the green plot are narrower, which highlights the advantage of the proposed approach. This is explained by the less accurate estimation of velocity during static phases (please refer to Figure 8). In fact, when the velocity is zero, or the magnetic field gradient is low (during static trajectories), observability issues of estimated states may arise, due to the term $\nabla B_{b}v_{b}$ in Equation (5) (the reader is invited to refer to [33] for more details about this issue). Thus, it is seen in Figure 8 that velocity tends to drift during static phases but quickly recovers afterwards. It is underlined here that the green plot of velocity performs better than the blue one, when the velocity is zero or close to it, and this is thanks to the added equation of the magnetic field gradient in the proposed approach.

Table 3 presents the RMSE, as well as the traveled distance error between the estimated position and the ground truth for the three studied approaches. As is the case for velocity, the best results are achieved when $\nabla B_{b}$ is filtered with the proposed EKF. This is clearly expected as the position is obtained from integrating the estimated velocity $\hat{v_{n}}$.

Despite the previously mentioned contribution in inertial velocity estimation, the obtained error results are still considered high if position reconstruction needs to be done, which is observed in Table 3. In fact, whether it is computed with or without the proposed model, $\hat{v_{n}}$ still suffers from some errors. One reason for these errors is the previously discussed observability issue during static trajectories (zero velocity and/or low values of the magnetic field gradient). Other errors are due to the different uncertainties considered in the simulation scenario—i.e., the approximations taken into account to extract the spatial derivatives ($T_{b}$ for instance), the linearization process of the EKF, the tuning of the process, and measurements covariances, etc. These errors lead to drifts if the position needs to be reconstructed, which is seen in Figure 9. Note also that a noise with a large standard deviation is applied on magnetometers measurements, in order to better highlight the contribution of filtering $\nabla B_{b}$. By looking back at Table 1, it is seen that the standard deviation of magnetometers’ noises is chosen equal to 0.03G. This value is over-evaluated. In fact, in commercialized magnetometers, it is found that the standard deviation of output noise can go up to 0.01G in some cases, but the most common range is around 0.005G. However, as the used data in this work are from [31], it is interesting to choose close parameters to theirs. In [46], the same authors indicate that the magnetometer has an added 0.1G standard deviation zero-mean Gaussian noise. This is considered very high compared to reality. For this reason, it is preferred to select a value that is between the previously found 0.01G and 0.1G, hence the choice of 0.03G in Table 1. Nevertheless, better velocity estimation results can be obtained in case the values of the different noises are lowered which, consequently, improves the position reconstruction.

#### 5.2.4. Zero-Velocity Update Results

**Figure 10:**
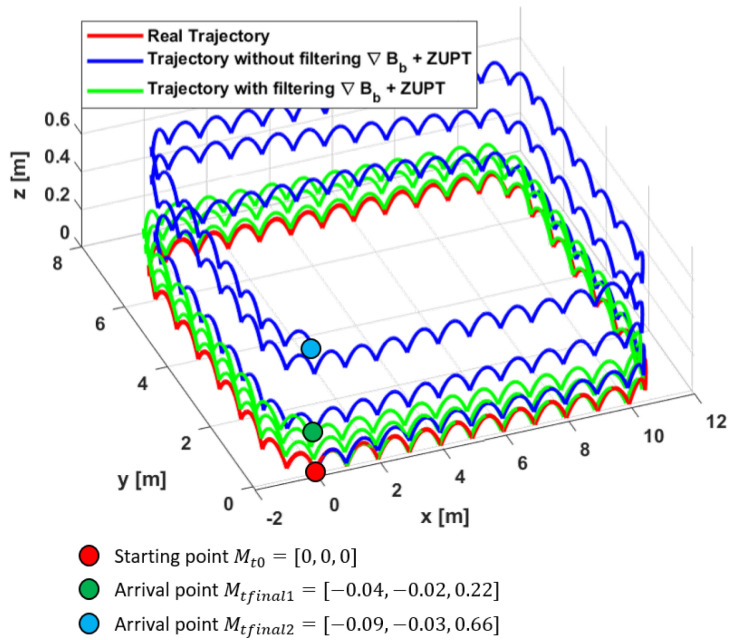
Trajectory reconstruction aided by ZUPT with and without filtering $\nabla B_{b}$.

For the different reasons stated above, the proposed magnetic field gradient-based EKF is combined with ZUPT (the red blocks in Figure 4), and the same comparisons are done on the position reconstruction as those in Figure 9. The pertinence of this approach on the velocity estimation in the case of foot-mounted applications is discussed in Section 4. By correcting the velocity estimate $\hat{v_{n}}$ with ZUPT, better position estimation results are obtained, and drifts on all three axes are almost entirely removed. In fact, Figure 10 shows that even when adding ZUPT, the proposed approach (with filtering $\nabla B_{b}$), still outperforms the case of when the filtering is not applied (use $\nabla B_{b}$ as a noisy input).

Note that the starting and arrival points for the ground truth trajectory are the same (red dot). It is observed that the arrival point of the green plot is closer to the ground truth one than the blue plot, which highlights the contribution of filtering $\nabla B_{b}$. It can also be seen from the points coordinates that the drift on the *z* axis is greatly reduced in the case of filtering $\nabla B_{b}$. In Table 4, a comparison between position RMSEs is displayed when ZUPT is added, which shows how the latter is reduced when $\nabla B_{b}$ is filtered. The obtained distance error with the proposed EKF after adding ZUPT also decreases from 1.22% of the total traveled distance to 0.41% with the proposed approach, which proves again the importance of filtering $\nabla B_{b}$.

## 6. Conclusions and Future Work

In this paper, the inertial velocity estimation was improved using a magnetic field gradient-based EKF. This was done by reducing noise from the magnetic field gradient, thanks to a newly introduced equation that better describes its dynamic. The proposed approach was then combined with ZUPT in order to estimate position in a foot-mounted application. Applying this approach on real experimental data is definitely the next step. Tuning the EKF covariance matrices with artificial intelligence-based approaches is also a topic that will be considered in future works.

## Figures and Tables

**Table 1:** Standard deviations of considered noises from datasheet of MTi module (Xsens) [44].

	Noise Standard Deviation
**Accelerometer $\;[{\mathrm{ms}}^{-2}]$**	**0.012**
**Gyroscope $\;[{\mathrm{rads}}^{-1}]$**	**0.0087**
**Magnetometers [G]**	**0.03**

**Table 2:** RMSE of the velocity estimation.

	$v_{n}\;\mathrm{RMSE}\;\left[{\mathrm{ms}}^{-1}\right]$
**Without filtering $\nabla B_{b}$** [27]	**0.37**
**With filtering $\nabla B_{b}$** in a primary EKF [34]	**0.29**
**With filtering $\nabla B_{b}$**	**0.27**

**Table 3:** Position estimation results.

	$M_{n}\;\mathrm{RMSE}\;\left[m\right]$	TraveledDistanceError[%]
**Without filtering $\nabla B_{b}$ [27]**	**31.60**	**36.77**
**With filtering $\nabla B_{b}$** in a primary EKF [34]	**25.18**	**21.60**
**With filtering $\nabla B_{b}$**	**20.88**	**19.50**

**Table 4:** Results of ZUPT-aided position estimation.

	$M_{n}\;\mathrm{RMSE}\;\left[m\right]$	TraveledDistanceError[%]
**Without filtering $\nabla B_{b}$ + ZUPT**	**0.26**	**1.22**
**Filtering $\nabla B_{b}$ in a primary EKF+ZUPT**	**0.14**	**0.88**
**With filtering $\nabla B_{b}$ + ZUPT**	**0.11**	**0.41**
